# MicroRNA miR-J1-5p as a potential Biomarker for JC Virus Infection in the Gastrointestinal Tract

**DOI:** 10.1371/journal.pone.0100036

**Published:** 2014-06-16

**Authors:** Alexander Link, Francesc Balaguer, Takeshi Nagasaka, C. Richard Boland, Ajay Goel

**Affiliations:** 1 Gastrointestinal Cancer Research Laboratory, Division of Gastroenterology, Baylor Research Institute, Baylor University Medical Center, Dallas, Texas, United States of America; 2 Department of Gastroenterology, Hepatology and Infectious Diseases, Otto-von-Guericke University, Magdeburg, Germany; 3 Department of Gastroenterology, Hospital Clinic, CIBEREHD, University of Barcelona, Spain; 4 Department of Gastroenterological Surgery and Surgical Oncology, Okayama University Graduate School of Medicine Dentistry and Pharmaceutical Sciences, Okayama, Japan; University of Kansas School of Medicine, United States of America

## Abstract

**Introduction:**

JC virus (JCV), a human polyomavirus that causes progressive multifocal leukoencephalopathy (PML), has been linked to colorectal cancer (CRC). However, determination of JCV infection and its role in carcinogenesis has been challenging, highlighting the need for better diagnostic strategies for this virus. JCV-specific microRNAs (miRNAs) were identified and shown to negatively regulate oncogenic JCV T-Ag. Herein, we determined the pattern of JCV miRNA expression in clinical specimens from healthy subjects and CRC patients.

**Material and Methods:**

JCV miRNA expression was validated in CRC cell lines transfected with the JCV T-Ag. Results were confirmed using CRC tissues that were expressed T-Ag. Expression of JCV-specific miR-J1-5p was measured in fresh stool samples from healthy volunteers, and samples from fecal occult blood test kits from healthy subject, and patients with colorectal neoplasms.

**Results:**

JCV miR-J1-5p was detected in JCV-transfected, but not vector-transfected, CRC cells, and was stable between cell passages. MiR-J1-5p was present in all six JCV T-Ag+ CRC samples. Surprisingly, JCV miRNA was detectable in all normal tissues, but the expression was much lower in CRC tissues. Similarly, miR-J1-5p expression was present in all fecal samples, but expression was lower in CRCs compared to controls or adenoma patients.

**Conclusion:**

JC virus-specific miR-J1-5p miRNA is a potential biomarker for viral infection, and the lower expression in patients with colonic neoplasia highlights its biological role regulating oncogenic T-Ag expression in CRC.

**Impact:**

JCV-specific miRNA is a candidate for the development of a non-invasive screening test, as well as therapeutic intervention for JCV-associated diseases.

## Introduction

JC virus (JCV) is a human polyomavirus known for over 40 years as a causative agent for a fatal demyelinating disease called progressive multifocal leukoencephalopathy (PML) [1], but more recently it has been implicated in carcinogenesis. In particular, it has been shown that infection with JCV promotes tumor development in hamsters and mice [2], [3]. JCV also shows significant sequence homology to monkey-polyomavirus SV40 and human BK virus [4]. JCV is a non-enveloped virus with double stranded, closed circular DNA 5.13 kb in length, and encodes six genes: 3 viral capsid proteins (VP1, VP2 and VP3), agnoprotein, small t-Ag and large T-Ag [5]. Analogous to SV-40, JCV T-Ag is one of the key components regulating the viral life cycle, as well as in virus-cell interaction. Current evidence highlights JCV T-Ag as the main oncogenic protein of JCV. JCV T-Ag is a multifunctional protein that has the ability to bind and break DNA, and has both helicase and ATPase activities [5]. Moreover, T-Ag can interact with key tumor suppressor proteins p53 and pRb through direct protein-protein binding, resulting in deregulation of cell cycle checkpoints and elimination of p53-mediated pro-apoptotic activity [5]. JCV T-Ag controls cellular proliferation by deregulating the Wnt signaling pathway through stabilization of β-catenin [6], [7] and its interaction with the IGF-IR signaling system for cellular transformation [8].

Although a direct link for JCV infection and human carcinogenesis remains elusive, a number of studies have demonstrated the presence of JCV genomic sequences and T-Ag expression in tumors, including brain and gastrointestinal malignancies such as colon, rectal, anal, gastric, and esophageal cancers [5]. Despite the fact that integration of JCV into the host genome has not been demonstrated, we recently showed concordant expression of JCV T-Ag in primary and metastatic CRC tumors [9]. In addition, the presence of JCV T-Ag expression exclusively in colonic adenoma and carcinoma cells, and a complete lack of expression in normal colon mucosa, further support the hypothesis of a potential oncogenic role for JCV in the early stages of this malignancy [10], [11].

The prevalence of JCV infection in humans has been a focus of multiple studies. Recent epidemiological analyses demonstrated the ubiquitous presence of JCV in human populations; however, the results varied substantially depending upon the method of virus detection. At present, detection of serum antibodies against JCV proteins is the most frequently used method to estimate the prevalence of this virus. It has been shown that the seroprevalence of JCV viral capsid protein, VP1, increases from 50–60% in children to 60–90% in adults, although other recent studies have reported a lower prevalence [12]–[17]. Nevertheless, since JCV infection in humans remains mostly inactive or latent, the clinical significance of seroprevalence is difficult to interpret, and the presence of preexisting antibodies does not necessarily indicate immunity.

Detection of viral DNA in different bodily fluids and tissues has been the focus of several studies. Our group showed that JCV T-Ag DNA was present in the upper and lower gastrointestinal tract in normal and neoplastic tissues in up to 90% of specimens [11], [18], [19]. Furthermore, conserved JCV DNA sequences have been frequently used as a biomarker for human fecal contamination with greater specificity than many types of gastrointestinal bacteria [20]. However, similar to seroprevalence, detection of JCV DNA in tissues does not translate into active infection in humans. On the other hand, JCV T-Ag expression has been considered a biomarker for active JCV infection, since it is assumed that the virus is actively transcribed and translated [11]. Functional studies of JCV T-Ag, as well as association studies of the presence of JCV T-Ag in GI tumors, are currently the key elements that support an oncogenic role for JCV in human cancers. However, JCV T-Ag expression is heterogeneous in most tissues and detectability is limited for two primary reasons: the lack of highly specific antibodies, and the inability of PCR to amplify genomic DNA with a high degree of specificity and reproducibility due to the supercoiled nature of the viral DNA [9], [11], [18].

MicroRNAs (miRNAs) are small non-coding transcripts that have been identified as a novel class of cellular molecules with high diagnostic, prognostic and therapeutic potential [21], [22]. One of the major biological functions of miRNAs is to regulate gene expression through direct binding to target mRNAs. In comparison to mRNA and DNA, miRNAs may serve as superior biomarkers because of their small size, higher stability (as these are protected from endogenous RNAase-mediated degradation), and the ease of detection in a variety of biological tissues and bodily fluids [23]–[25]. It has been shown that JCV encodes a pre-miRNA that is processed into two unique miRNAs (JCV-specific miR-J1-5p and miR-J1-3p) during the late phase of infection [26]. Both miRNAs are capable of downregulating early phase protein T-Ag that controls the life cycle of the virus [26]; however, the translational significance and clinical value of these miRNAs remains to be determined.

The goals of the present study were to evaluate the presence, and pattern of JCV-specific miRNA in colonic tissues and feces, in an attempt to determine if this miRNA can serve as a biomarker for JCV infection in humans. To address this question, we systematically evaluated JCV-miRNA expression in a panel of CRC cell lines and subsequently generated stable clones of JCV T-Ag-expressing cells. Next, we evaluated the expression of miR-J1-5p in CRC tissues and stool samples from healthy subjects and patients with colorectal adenomas and cancers. Results from this preliminary study suggest a potential role of JCV-specific miR-J1-5p as a novel biomarker for JCV infection in the colon.

## Materials and Methods

### Cell Culture and Transfection

Two human CRC cell lines, HCT116 (with microsatellite instability or MSI) and WiDr (microsatellite stable or MSS) were obtained from the American Type Culture Collection (ATCC, Manassas, VA). All cells were tested and authenticated in our laboratory every 6 months using known genetic and epigenetic marks. The cells were cultured in IMDM (Life Technologies, Carlsbad, CA) and supplemented with 10% fetal bovine serum (Invitrogen) with 5% CO_2_ at 37°C. The cells were transfected with full-length JCV-early transcript coding region cloned into a pCR3 vector (called JCV_E_, kindly provided by Dr. Richard Frisque of the Pennsylvania State University, University Park, PA) or an empty vector control as previously described [9]. Briefly, transfection was performed with Effectene (QIAGEN, Valencia, CA) using standard protocol and stable clones were selected following treatment with 600–1000 µg/ml of G418 (Geneticin) (Sigma-Aldrich, St. Louis, Mo). The selected cells were maintained in 200 µg/ml of G418 prior to further analyses and JCV T-Ag expression was regularly monitored by RT-PCR and Western immunoblotting.

### RNA Isolation

Total RNA, including miRNAs, was isolated from frozen cell pellets, frozen tissues and stool samples using the miRNeasy Mini kit (QIAGEN) according to the manufacturer’s instructions with some modifications for stool samples as previously described [24]. RecoverAll extraction kit (Life Technologies, Inc., Carlsbad, CA) was used to isolate total RNA from 5-µm formalin-fixed, paraffin-embedded (FFPE) sections according to the manufacturer’s instructions [27]. RNA quality and quantity were assessed using A230/A260 ratios.

### RNA Analyses using Reverse Transcriptase PCR

To analyze the JCV T-Ag mRNA expression, total RNA was reverse transcribed to cDNA from 1 µg of total RNA using random hexamers and the Advantage RT-for-PCR Kit (Clontech Laboratories, Inc., Mountain View, CA). JEX primers were used to specifically detect JCV T-Ag sequences as previously described [9].

### Microrna Quantification by Real-Time RT-PCR

Quantification of miRNA expression was performed using either TaqMan miRNA Assays (Applied Biosystems, Grand Island, NY) or SYBRgreen (Life Technologies, Carlsbad, CA) as previously described [24]. Briefly, ∼20 ng of RNA was reverse transcribed and real-time PCR was performed using an Applied Biosystems 7300 Sequence detection system. Mean values (n = 3) were used to calculate the fold difference using the standard 2^−ΔΔCt^ or 2^−ΔCt^ methods. Normalization was performed using RNU6b levels for fresh frozen cells or miR-16 levels for FFPE tissues, and mean miR-16 and miR-26b concentrations for fecal specimens [24], [27].

### Western Immunoblotting

To test JCV T-Ag protein expression in cancer cells, western immunoblotting was performed as described previously [9]. Briefly, cells were lysed in a lysis buffer (0.5 M Tris pH 6.8, 80% glycerol, 1.2 g sodium dodecyl sulfate, and 0.5 M EDTA) and heat-denatured. Electrophoresis was performed on 10% denaturing polyacrylamide gels and proteins were transferred to PVDF membranes (Amersham Hybond-P, GE Healthcare Life Sciences, Piscataway, NJ). The blots were probed with mouse monoclonal antibody cocktail against JCV T-Ag (clones PAb2003, PAb2023, PAb20024, PAb2030, PAb2001, PAb2000 kindly provided by Dr. R. Frisque [28], [29]) at a 1∶50 dilution and mouse anti-β-actin antibody (Clone AC-15; at dilution of 1∶32,000). Horseradish peroxidase-conjugated IgG secondary antibody was used for signal detection prior to exposure of the membranes to the ECL reagent (Amersham GE Healthcare) and final visualization using the Storm imaging system (Nikon, Melville, NY).

### Immunohistochemistry

Six 4 µm FFPE CRC tissue sections were stained for JCV T-Ag as previously described [11]. Slides were incubated overnight with primary mouse monoclonal antibody against SV40 T-Ag that cross reacts with JCV T-Ag (clone PAb416, 1∶40 dilution, Calbiochem, Billerica, MA) followed by incubation in Dako EnVision labeled polymer (Dako North America, Inc., Carpinteria, CA) [10], [11]. Staining was developed by reaction with diaminobenzide and counterstained with hematoxylin. JCV-inoculated hamster brain tumor tissue slides (kindly provided by Dr. Kamel Khalili, Temple University, Philadelphia, PA) were used as a positive control for JCV-specific nuclear staining, as described previously [30].

### Tissues Specimens

Six FFPE CRC tissues from patients with sporadic CRC treated by physicians associated with the Baylor University Medical Center, Dallas, and twenty-one paired samples (tumor and normal mucosa) from patients with sporadic CRC were consecutively collected at Okayama University Hospital, Okayama, Japan. Written informed consent was obtained from all patients under protocols approved by the institutional review boards (IRBs), or de-identified tissues were used under approval from the IRBs of Baylor University Medical Center, Dallas and Okayama University, Japan. Clinical and demographical data of the patients are presented in Table 1.

**Table 1:** Clinico-pathological characteristics of patients and healthy volunteers in the study.

Specimen	Tissue	FFPE	Feces	FOBT/Feces		
Disease	CRC	CRC	Volunteers	Controls	Adenoma	CRC
Number (n)	21*	6	8	10	9	10
Sex						
Women	8	3	4	3	3	7
Men	12	3	4	7	6	3
Age (mean ± SD)	64.4±10.8	60.3±4.5	28.8±6.6	72.7±10.5	64.7±14.4	63.2±10.8
Adenoma						
Advanced					3	
Non-advanced					6	
Differentiation						
Moderate	14					
Well diff. and mucinous	5					
Poor	1					
Localization						
Proximal	12	2			4	5
Distal	8	4			5	5
Cecum	1					-
Ascending	10				4	5
Transverse	1	2			1	-
Descending		1			1	2
Sigmoid	3	3			3	2
Rectum	5				1	1
UICC-Stage						
I	2	1				1
II	4	4				4
III	8					2
IV	6	1				3

*Clinical characteristics of one patient was incomplete.

### Stool Specimens

To test the feasibility and reproducibility of the measurements, we prospectively obtained fresh stool samples from 8 healthy individuals (4 males and 4 females, mean age 28.9 (21–41) years). Once collected, all samples were stored at −80°C and RNA isolation was performed within 2–3 weeks. To test the JCV miRNA expression in patients with colorectal neoplasia, a total of 29 stool specimens were randomly selected from a larger collection of fecal samples that were collected in FOBT kits (Eiken Chemical Co., Bunkyo-ku, Japan). These fecal samples were obtained from 10 individuals with normal colonoscopies, 9 patients with advanced or non-advanced colonic adenomas, and 10 patients with CRC as previously described [24]. Written informed consent was obtained from all healthy volunteers and patients, and the study protocol was approved by the IRBs. Clinical and demographical data of the patients were published previously [24].

### Statistical Analysis

Data analyses were performed with GraphPad Prism 4.0 software (San Diego, CA, USA). The differences between two groups were analyzed using Student’s t-tests, and between more than two groups using ANOVA with Bonferroni’s multiple comparisons *post hoc* test. Correlation analyses were performed using Pearsońs test and logarithmic regression was used to calculate the R^2^ and to generate the equation of the slope. Two sided p-values of <0.05 were considered statistically significant.

## Results

### JCV-specific miRNA Expression in CRC Cell Lines

**Figure 1:**
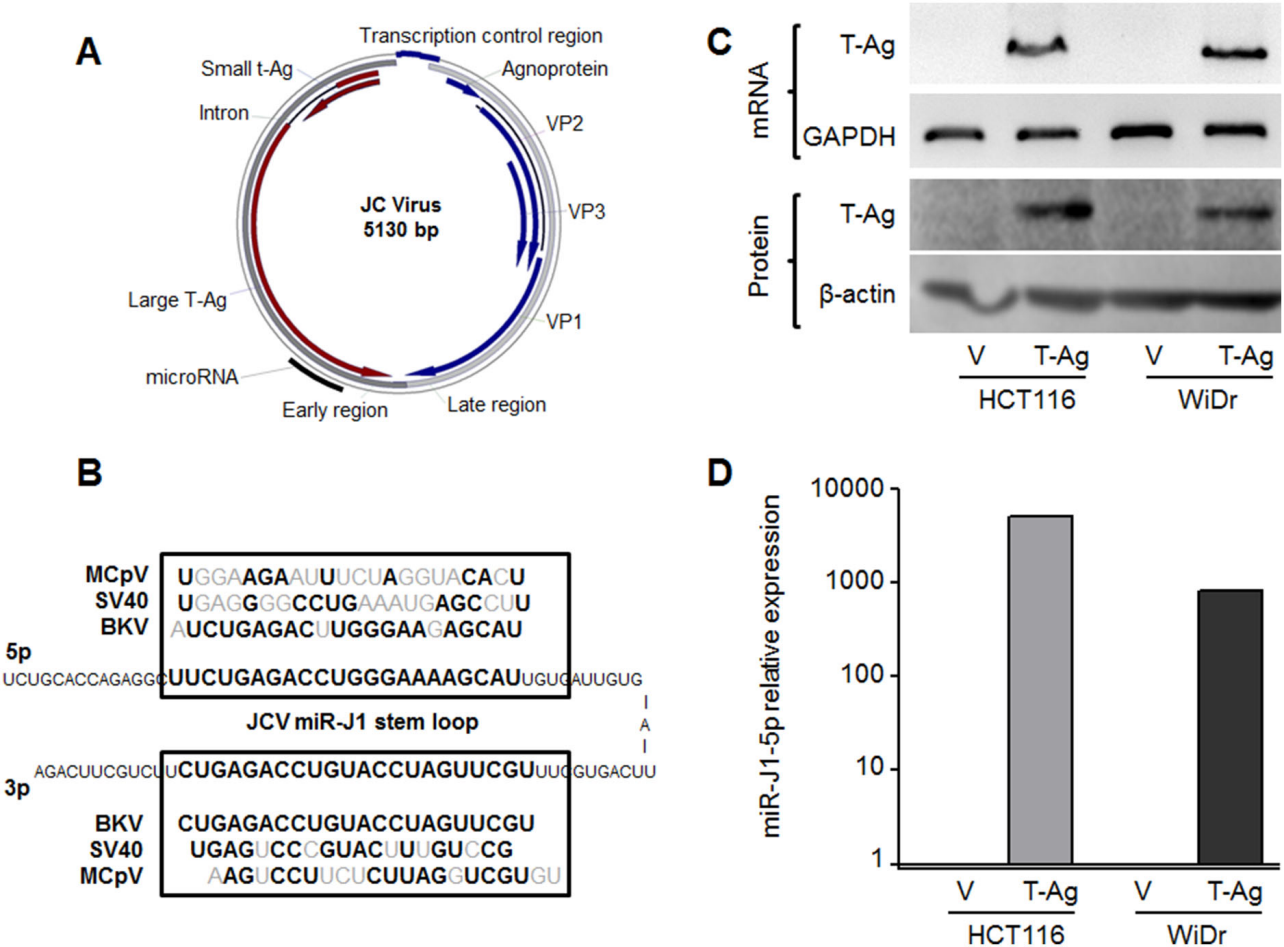
JCV miRNA sequence and detection. (A) Schematic presentation of the JCV genome. The black circle marks the transcript location of the JCV miR-J1 stem loop. (B) JCV miR-J1-5p and -3p sequences are compared to the Merkel Cell Polyomavirus-, SV40- and BK virus-miRNA sequences. (C & D) CRC cells were transfected in vitro with a JCVT-Ag-E plasmid, and JCV T-Ag message and miRNA expression were analyzed. In C, GAPDH and β-actin were used as loading controls for mRNA and protein expression, respectively. (D) Vector transfected cells showed no detectable miR-J1-5p expression, while JCV miR-J1-5p expression was high in transfected cells. To measure the expression of miR-J1-5p, expression in the vector was set to a Ct-value of 40, and 2^−ΔΔCt^ values were calculated using RNU6b for normalization.

The JCV-specific precursor and miR-J1 genes are located within the JCV T-Ag sequence and demonstrate a high degree of homology to other polyomaviruses such as SV40, BKV and Merkel Cell polyomaviruses (**Figure 1A**) [26]. Comparison of the miRNA sequences for these different polyomaviruses revealed that the miR-J1-5p sequence is particularly specific for JCV, while the miR-J1-3p sequence is identical to the sequence of BKV miR-B1-3p (**Figure 1B**). To eliminate any potential problems with cross reactivity to BKV miR-B1-3p in human samples, we focused our study on JCV-miR-J1-5p. At first, we ascertained whether JCV-miR-J1-5p could be identified in cultured cells, as some previous studies failed to detect JCV T-Ag mRNA or protein expression in CRC cell lines [9], [31]. Using probe-based customized TaqMan primers, we did not observe miR-J1-5p expression in the parental HCT116 and WiDr cell lines, suggesting that there was no cross-reactivity with any other abundantly expressed miRNAs in the CRC cell lines (data not shown).

### Induction of JCV miRNA Expression In vitro

To evaluate whether transfection of the JCV T-Ag transcript region into CRC cells would induce JCV miRNA expression that is detected with a probe for miR-J1-5p, we established a cell model. We stably transfected CRC cells HCT116 and WiDr with the JCV early transcript region-JCVe, which includes the JCV precursor RNA for JCV-miR-J1 expression (**Figure 1A**). The stable transfection with JCVe was confirmed by measurement of JCV-T-Ag mRNA and protein shown in **Figure 1C**. Compared to the cells transfected with empty vector, JCVe-transfected cells, which includes the JCV-miR-J1 coding sequence, demonstrated >1000-fold increase in expression of JCV-miR-J1-5p (**Figure 1D**). Analyses of independent passages of CRC cells showed comparable expression of miR-J1-5p and -3p in several clones (data not shown) confirming specific and stable expression of JCV-miR-J1-5p expression in the cell model we developed.

### JCV miRNA Detection in JCV T-Ag+ CRC Tissues

**Figure 2:**
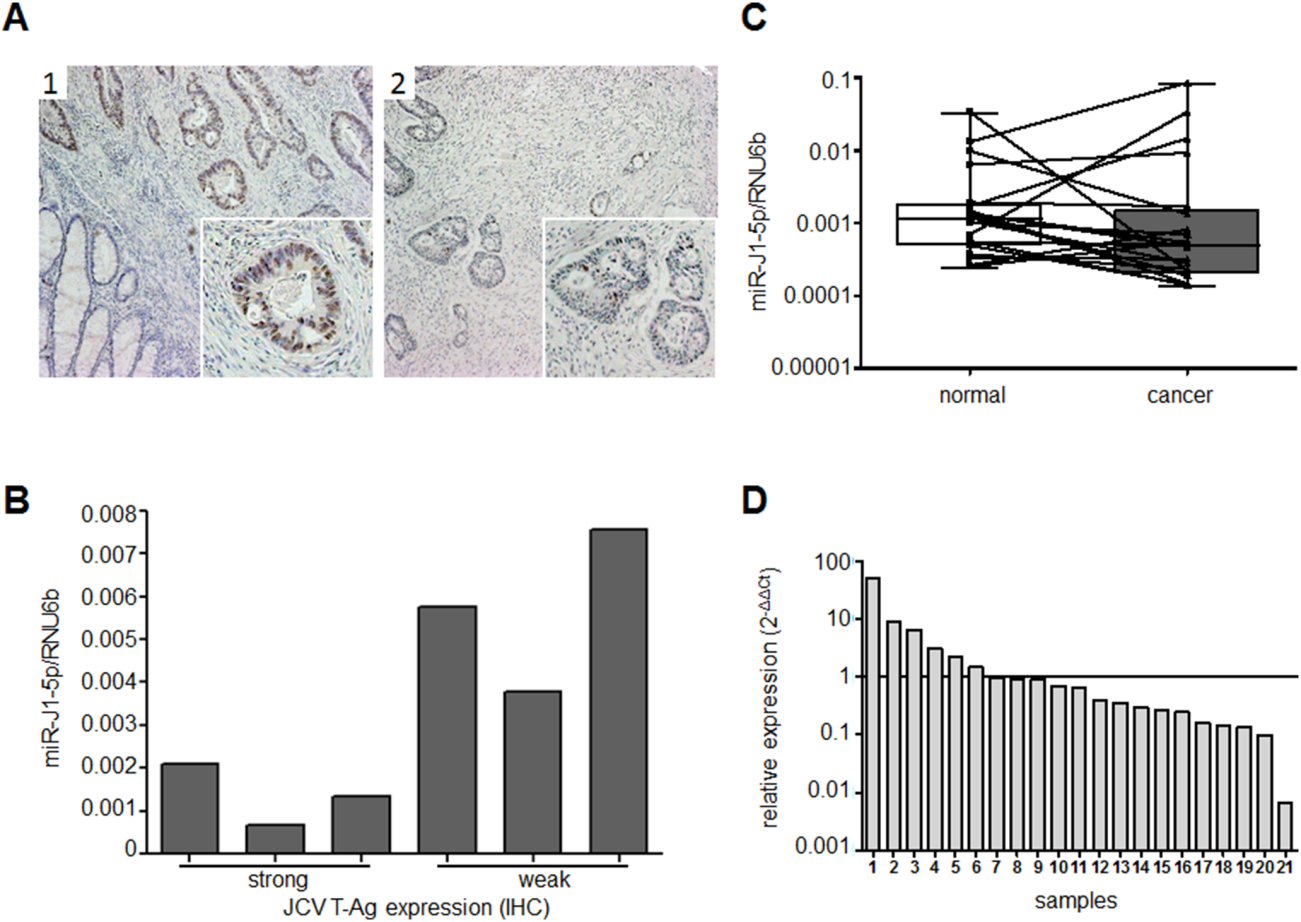
JCV miR-J1-5p detection in CRC patient tissues. (A) Six FFPE tissue specimens were stained for JCV T-Ag expression to ensure the active presence of JCV in CRC tissues. Figure A1 shows a representative image for strong, and A2 shows weak JCV-T-Ag protein expression. (B) JCV miR-J1-5p expression was evaluated in each of 3 samples with strong and weak JCV T-Ag expression. Normalization of miR-J1-5p expression in FFPE tissues was performed using miR-16, as previously validated. (C & D) miR-J1-5p expression was evaluated in paired normal colonic mucosa and CRC fresh frozen tissues from 21 patients with CRC. In C, miR-J1-5p expression is shown for paired normal colonic mucosa and CRC tissues. miRNA expression is shown as 2^−ΔCt^ normalized to RNU6b expression. (E) miR-J1-5p expression in CRC tissues is shown correlated with miR-J1-5p expression in normal colon mucosa. The results are presented as 2^−ΔΔCt^ normalized to RNU6b and matching normal colonic mucosa, and the values are sorted in descending order. From a total of 21 CRC tissues samples, 12 samples (below the line) showed lower, and 6 samples (above the line) higher miR-J1-5p expression in CRC tissues compared to normal mucosa.

Having shown that JCV miRNA may be specifically detected in JCV-transfected CRC cells in culture, we determined whether JCV miR-J1-5p is expressed in human CRC tissues, particularly ones with detectable JCV DNA and T-Ag expression. We used six FFPE tissues: three samples with strong JCV T-Ag expression (**Figure 2A**, representative image 1), and three with low expression (**Figure 2A**, representative image 2). All six of the tissue samples showed the presence of JCV miR-J1-5p, although the amounts were variable (**Figure 2B**). When JCV T-Ag expression was compared with JCV miR-J1-5p expression, higher expression of JCV miR-J1-5p was seen in tissues with lower or weaker JCV T-Ag expression (**Figure 2B** **, 4–6**) compared to those with strong T-Ag expression (**Figure 2B** **,** **1** **–** **3**).

**Figure 3:**
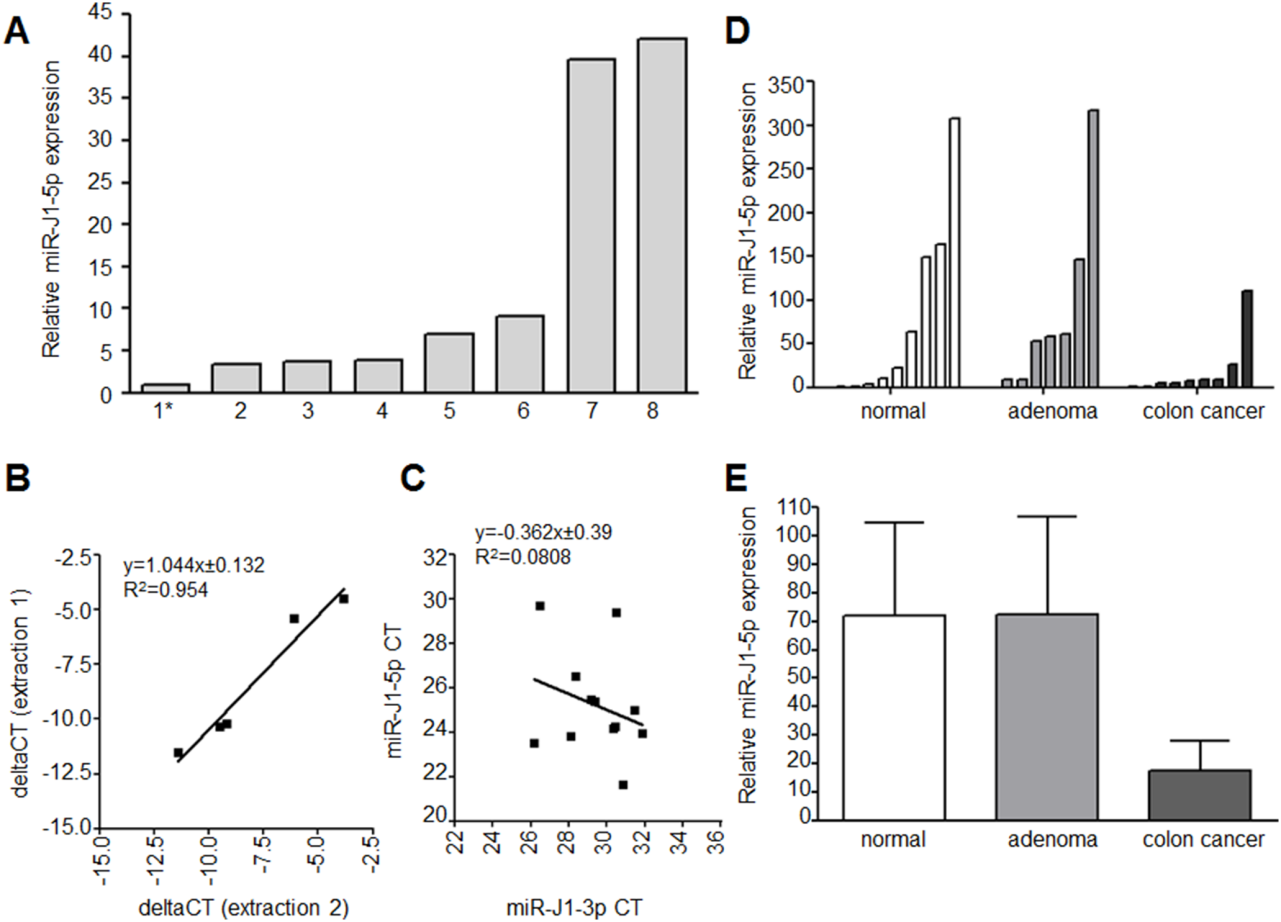
JCV miR-J1-5p detection in feces. (A) To test whether JCV miRNA is present in stool, we extracted total RNA from stool samples and performed TaqMan based miRNA expression analyses. Expression of miR-J1-5p was normalized to mean miR-16 and -26b levels and further adjusted to the sample with the lowest miR-J1-5p expression level (1*). (B) To test the reproducibility of miRNA detection, we performed independent RNA extraction from the same samples in the subset of fecal samples from healthy subjects (n = 5). The samples were normalized to mean miR-16 and -26b expression. (C) Concomitant expression analyses of miR-J1-5p and -3p showed no correlation with JCV miRNA expression, arguing for potential cross-reactivity with BKV microRNA. (D&E) To measure JCV miR-J1-5p expression in feces from CRC patients, miR-J1-5p was analyzed by TaqMan PCR in 29 FOBT specimens from patients without and with colorectal neoplasia. Fold-expression was calculated using the 2^−ΔCt^ method normalized to mean miR-16 and -26b expression. D Represents the single sample values and E the mean values ± SD.

### JCV miRNA Detection in Paired Normal and CRC Tissues

To determine if JCV miR-J1-5p is expressed in normal colon mucosa as well as CRC tissues, we studied paired normal colon mucosa and CRC tissues. Interestingly, both normal mucosa and CRC tissues from CRC patients expressed JCV miR-J1-5p, although there was considerable variation in the amounts (**Figure 2C**). In a comparison of normal versus malignant tissues, we found that the majority of CRC tissues exhibited lower expression of miR-J1-5p (62%) compared to normal mucosa from the same patients (**Figure 2E**), although the data did not reach statistical significance (p = 0.34).

### JCV miRNA is Present in Stool Samples from Healthy Volunteers

As miRNAs can be detected in feces, we determined whether JCV miRNA could be detected in feces as well. Using total RNA isolated from freshly collected feces from eight healthy volunteers, we found that miR-J1-5p was detectable at low levels in feces from all eight samples (**Figure 3A**). Following adjustment of miR-J1-5p expression to the sample with lowest expression, we observed >40-fold expression differences among the healthy subjects. To exclude potential methodological variations in these results, we performed miR-J1-5p expression analyses in matching samples using independently extracted fecal total RNA and found a high degree of reproducibility for the detection of miR-J1-5p (r^2^ = 0.9542, p = 0.0042) (**Figure 3B**).

Since miR-J1-5p and miR-J1-3p are processed from the same precursor miRNA, we determined whether our results were a consequence of changes in precursor miRNA expression. Therefore, we determined whether miR-J1-5p expression correlated with miR-J1-3p expression. We found no significant correlation between miR-J1-5p and -3p levels in the feces of healthy subjects, suggesting that these two miRNAs are either regulated independently, or the detection of JCV-miR-J1-3p may be biased because of homology to miR-J1-3p to BKV (**Figure 3C**).

### JCV miRNA Expression in Feces of Patients with Colonic Neoplasia

Based on the detection of miR-J1-5p in normal colon mucosa and CRC tissues, we extended our analyses to compare miR-J1-5p levels in fecal specimens from patients with no neoplasia, adenomas, and CRC (**Figure 3D and E**). As with our previous results, we found lower expression of mir-J1-5p in stool samples from patients with CRCs compared to subjects without neoplasia or subjects with adenomas (miR-J1-5p/RNU6b relative expression mean ± SD: 17.3±33.6 vs. 72.5±102.8 and 72.1±103.5, for CRC, adenomas, and controls, respectively). Nevertheless, this difference did not reach statistical significance due to the variability among the samples and the small number of fecal samples analyzed (p = 0.129).

## Discussion

In this study, our goal was to evaluate the feasibility of using JCV-specific miRNA as a biomarker for JCV infection in the gastrointestinal tract, and possibly as a marker for colorectal neoplasia. We demonstrated that JCV miR-J1-5p expression could be induced following transfection with the JCV T-Ag early sequence in cultured CRC cells, but not in control or vector transfected cells. Following systematic analyses of miRNA expression in normal colonic mucosa, CRC tissues, and feces from healthy and patients with colon neoplasia, we showed that JCV miR-J1-5p has a specific expression pattern, which differs from the pattern of the precursor RNA for JCV-miR-J1 and the JCV T-Ag protein. Of importance, we found that miR-J1-5p was expressed in all colonic tissues. Interestingly, there was a trend for lower expression of miR-J1-5p in colonic tissues and fecal samples from CRC patients compared to normal patients or patients with adenomas. These results provide the novel observation that miR-J1-5p may serve as a specific biomarker for JCV infection in the gastrointestinal tract.

In order to improve our current understanding of JCV-related diseases, it is crucial to better understand all steps of the viral life cycle. In addition to the lack of reliable diagnostic and prognostic biomarkers for JCV, we do not have effective therapies to treat JCV-related disease. There are several limitations in the currently used methods to diagnose JCV infection, such as JCV DNA detection, serological testing, and analyses of JCV T-Ag expression. Since its discovery, the use of miRNA expression has become a novel and promising tool for biomarker research. Multiple studies have highlighted the unprecedented stability, and ease of measurement of miRNAs. In particular, several studies have explored the potential for use of viral miRNA expression as a specific surrogate for the presence of viral infection [32]. In a pivotal study, Seo et al. showed that JCV produces 2 evolutionarily conserved miRNAs, miR-J1-5p and miR-J1-3p, and that JCV-specific miR-J1-5p is detectable in brain tissues of PML patients [26]. Gee et al. used a similar approach to investigate whether SV40-specific viral miRNA may be detectable in pleural mesotheliomas; however, they failed to detect viral miRNAs in tumor tissues [33]. Indeed, we focused our study mostly on the miR-J1-5p because miR-J1-3p has complete sequence homology to BKV, and we wanted to ensure that our study results were more specifically representative of JCV-specific miR-J1-5p from a biomarker standpoint.

To investigate the distribution of JCV miRNA in humans, we used CRC as a model for our study [5]. After determining the feasibility and specificity of detecting JCV miR-J1-5p in JCV T-Ag transfected cells, we systematically evaluated different types of specimens for the presence of miR-J1-5p. Interestingly, we found that JCV miR-J1-5p was present both in normal human colonic mucosa, as well as in neoplastic colonic tissues. Additionally, miR-J1-5p was present in feces from both healthy subjects and patients with CRC. In both tissues and feces, there was a trend for miR-J1-5p to be lower in samples from patients with CRC. The presence of JCV miRNA in feces was expected, as it was described previously in the gastrointestinal tract in up to 90% of healthy subjects [11], [18], [19].

The role of viral miRNA in the establishment of latent infection has been proposed by Seo et al. [26]. The authors demonstrated that JCV miRNAs function to promote latent infection through a decrease in the expression of large T-Ag. In study from Baumann et al. [34], it was shown that a JCV miRNA-mediated decrease in ULBP3 expression resulted in fewer virus-infected cells being killed by natural killer cells. The authors postulated that JCV miRNAs may play a role in the process of promoting latent infection by allowing the virus to escape detection by the immune system. Based on these results, one can hypothesize that JCV latent infection (associated with the expression of JCV miRNAs) could provide some transient advantage to colon mucosa with early neoplastic changes by promoting escape from immune detection, while this immunological defense may not be needed during the late stages of carcinogenesis, where we found decreased miR-J1-5p expression. Indeed, our results correlate with those of Seo et al. [26], as we found some evidence for lower expression of miR-J1-5p in CRC tissues, which correlated inversely with increased levels of JCV T-Ag expression. Further systematic studies will be needed to address the question whether this tumor-associated increase in JCV T-Ag expression is a cause or consequence of reduced miR-J1 expression.

Several epidemiological studies have demonstrated that JCV may be present ubiquitously in the human population. The presence of JCV in the gastrointestinal tract and in urban sewage suggests a fecal-oral route for widespread dissemination. This is consistent with our observation that JCV miRNA was present in all fecal samples from healthy subjects. Assuming that miR-J1-5p may be expressed only in human cells using human miRNA-machinery (Drosha, Dicer etc.), the detection of this miRNA in feces further suggests not just the simple presence of JCV in feces, but additionally active viral replication with an inadequate immunological response. This is an area that needs further study, as our healthy subjects exhibited great variability in the levels of JCV miRNA in their feces, with >40-fold differences among participants.

Overall, our study is one of the first to address the JCV miRNA expression in colonic tissues and feces. Although, we have used various tissue sources (normal mucosa, cancer tissues, feces from healthy subject and feces from patients with colon neoplasia), further systematic studies are needed to correlate analyses of DNA and T-Ag expression in larger number of samples. Since JCV T-Ag has been detected not only in CRC, but also in gastric and esophageal cancers, future studies will need to address the miR-J1-5p expression and function in these various gastrointestinal malignancies.

In summary, we demonstrate in this pilot study that JCV miRNAs can be easily and reproducibly detected in human colonic tissues and feces. There was a trend for JCV miRNA to exhibit lower expression in CRCs and feces from patients with CRC. In order to better address the diagnostic potential for the use of JCV miRNA as a diagnostic biomarker for infection, further studies need to be performed systematically on many more subjects and patients including to evaluate the effect on prognosis or survival of CRC patients. Because of the sequence specificity, JCV-miR-J1-5p may serve as potential biomarker for JCV infection and offer a novel approach for the study of JCV biology and its role in colorectal neoplasia.
